# Contrast-enhanced transrectal ultrasound for prediction of prostate cancer aggressiveness: The role of normal peripheral zone time-intensity curves

**DOI:** 10.1038/srep38643

**Published:** 2016-12-08

**Authors:** Hui Huang, Zheng-Qiu Zhu, Zheng-Guo Zhou, Ling-Shan Chen, Ming Zhao, Yang Zhang, Hong-Bo Li, Li-Ping Yin

**Affiliations:** 1 Department of Ultrasound, Affiliated Hospital of Nanjing University of TCM, Nanjing 210029, China; 2 Department of Radiology, Affiliated Hospital of Nanjing University of TCM, Nanjing 210029, China; 3 Department of Pathology, Affiliated Hospital of Nanjing University of TCM, Nanjing 210029, China; 4 Department of Urology, Affiliated Hospital of Nanjing University of TCM, Nanjing 210029, China

## Abstract

To assess the role of time-intensity curves (TICs) of the normal peripheral zone (PZ) in the identification of biopsy-proven prostate nodules using contrast-enhanced transrectal ultrasound (CETRUS). This study included 132 patients with 134 prostate PZ nodules. Arrival time (AT), peak intensity (PI), mean transit time (MTT), area under the curve (AUC), time from peak to one half (TPH), wash in slope (WIS) and time to peak (TTP) were analyzed using multivariate linear logistic regression and receiver operating characteristic (ROC) curves to assess whether combining nodule TICs with normal PZ TICs improved the prediction of prostate cancer (PCa) aggressiveness. The PI, AUC (*p* < 0.001 for both), MTT and TPH (*p* = 0.011 and 0.040 respectively) values of the malignant nodules were significantly higher than those of the benign nodules. Incorporating the PI and AUC values (both, *p* < 0.001) of the normal PZ TIC, but not the MTT and TPH values (*p* = 0.076 and 0.159 res*p*ectively), significantly improved the AUC for prediction of malignancy (PI: 0.784–0.923; AUC: 0.758–0.891) and assessment of cancer aggressiveness (*p* < 0.001). Thus, all these findings indicate that incorporating normal PZ TICs with nodule TICs in CETRUS readings can improve the diagnostic accuracy for PCa and cancer aggressiveness assessment.

Prostate cancer (PCa) is the second most common malignant tumour that affects the male population^1^. Timely diagnosis of PCa can ensure efficient management of the disease and improve active surveillance in patients with and without clinically significant cancers^2^. Consequently, the precise evaluation of prostate nodules is vital.

Contrast-enhanced transrectal ultrasound (CETRUS) has been used widely to enhance the visualization of perfusion changes related to PCa, and it is regarded as a promising tool for evaluating malignant prostate nodules^3^. In clinical practice, PCa is confirmed or excluded by transrectal ultrasound-targeted biopsy, based on the results of digital rectal examination or serum prostate-specific antigen (PSA) levels^4^, and perfusion analysis by CETRUS^5^. However, recent findings have demonstrated that CETRUS is of limited value for evaluating the aggressiveness of PCa, owing to its varying sensitivity and specificity^5^,^6^.

PCa generally exhibits altered angiogenic microvascular structures and blood flow patterns, which characterize the aggressiveness of this cancer and form the basis of diagnosis based on CETRUS^7^,^8^. However, the flow pattern signals of this malignancy are often subtle and disappear within seconds; the efficiency of this method is therefore highly user dependent. Therefore, quantitative techniques that extract perfusion parameters to distinguish between malignant and benign tissue are being developed from CETRUS recordings: one of these techniques is the creation of time-intensity curves (TICs). TICs include many crucial parameters, such as arrival time (AT)^9^, mean transit time (MTT) and rise time^10^, time to peak (TTP) and peak intensity (PI)^9^, among others. These quantitative indices are reported to have high diagnostic accuracy and lesser user dependency. However, the discriminative power of these indices depends in part on the “background” variability of tissue heterogeneity, which when eliminated improves the diagnostic accuracy of magnetic resonance imaging (MRI) for PCa detection^11^. However, to date, although CETRUS is a commonly used modality for the diagnosis of malignant prostate nodules, TICs of the normal peripheral zone (PZ) are still rarely taken into account.

Therefore, the aim of this study was to evaluate the role of normal PZ TICs for the detection of PCa and assessment of cancer aggressiveness, and to determine whether the diagnostic accuracy improves significantly, compared to the biopsy findings, when normal PZ quantitative parameters are incorporated.

## Material and Methods

This study was approved by the ethics committee of the Jiangsu Province Hospital of Traditional Chinese Medicine, and the methods were carried out in accordance with the relevant guidelines. Written informed consent was obtained from all patients.

### Patients

Between May 2014 and March 2016, 132 patients with abnormal digital rectal examination findings and/or elevated serum PSA levels (≥4 ng/ml and ≤10 ng/ml) who had not previously undergone biopsy were enrolled in this study. All the patients underwent CETRUS, which was performed by an experienced operator, and all the CETRUS data were independently reviewed frame by frame on the scanner by two other experienced investigators who were blinded to the greyscale imaging and pathological results. Then, a systematic twelve-core transrectal ultrasound-guided prostate biopsy and two-core targeted biopsy were performed based on the abnormal sonography findings by an experienced operator from the Department of Urology, who was assisted by the CETRUS operator mentioned earlier.

### CETRUS procedure

**Figure 1:**
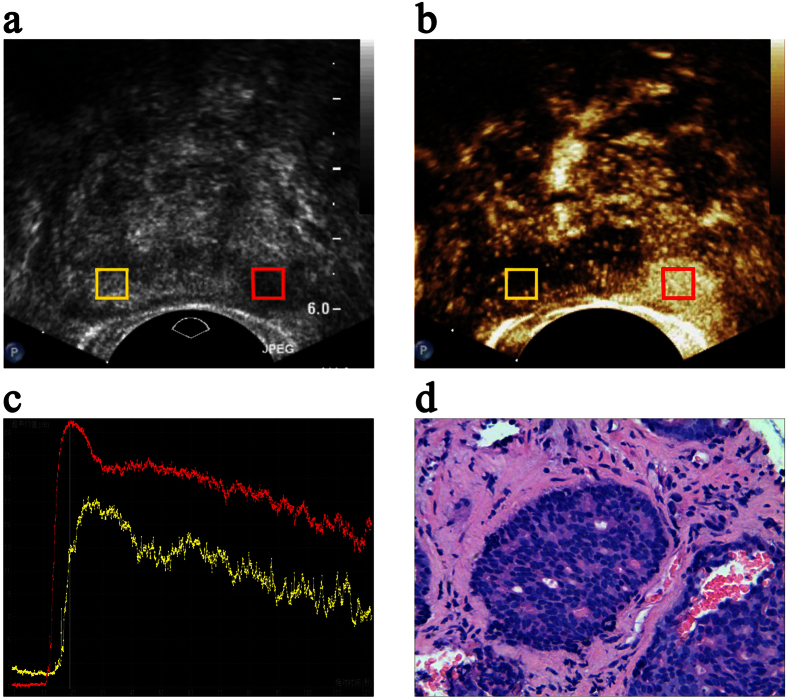
The region of interest (ROI) settings, time-intensity curve (TIC) analysis and oncological outcomes. Transverse (**a**) greyscale and (**b**) contrast-enhanced transrectal ultrasound mapping of the prostate in a 67-year-old patient. The red box indicates the ROI placed in the nodule lesion, and the yellow box indicates the corresponding ROI placed in the normal peripheral zone (PZ). (**c**) The red and yellow lines in the TICs represent the perfusion characteristics of the ROIs of the nodules and the normal PZ, respectively. (**d**) The oncological outcomes following biopsy show an adenocarcinoma.

Each patient was evaluated using ultrasonography at the baseline and again during intravenous infusion of sulphur hexafluoride microbubbles (SonoVue; Bracco, Milan, Italy) (Fig. 1a,b). An IU-22 ultrasound system (Philips, Amsterdam, The Netherlands) with a transrectal end-fire transducer (c8-4v) was used. Normal greyscale imaging was performed with a probe frequency of 4 to 8 MHz and a dynamic range of 55 dB. For colour Doppler ultrasonography, the probe frequency was 6 MHz, and the colour Doppler gain was adjusted to maximize signal but eliminate colour noise from the tissue of the prostate. The colour Doppler window was set to include the entire gland. During the contrast-enhanced ultrasound examinations, a fast bolus injection of 2.4 ml SonoVue was administered intravenously; this was followed by administration of 5 ml of normal saline flush. The scanner was set in the contrast pulse-sequencing mode with a probe frequency of 8 MHz. The acoustic power was set at a mechanical index of 0.13, and the dynamic range was fixed at 55 dB. The transverse plane of the sonographic abnormality was chosen for contrast imaging. In patients with no suspicious baseline ultrasonography results, the most hypervascular plane on colour Doppler images was chosen. The entire examination was saved in a Digital Imaging and Communications in Medicine format and transmitted to a workstation for further analysis (Fig. 1c).

### Image analysis

All CETRUS data were analyzed on the workstation using the QLAB quantification software (Philips) by a sonographer who was blinded to all the clinical and pathological information (Fig. 1c). Considering that the enhancement characteristics of the PZ lesions were completely different from those of the transition zone lesions, which may reflect the hypervascularity of the normal inner gland and coexisting benign prostate hyperplasia, only the PZ was evaluated in this study. Therefore, duplicated regions of interest (ROIs) were drawn in the targeted biopsy and normal PZ site on contrast ultrasonographic images, and the diameters were set to approximately 5 mm. The TICs were reconstructed for each ROI, and then the relative quantitative parameters, which depicted the features of prostate tissue infusion in the ROI that were observed after time zero, were measured by three well-trained observers. The average of all the measurements was calculated.

### Analysis of oncological outcomes

Biopsy specimens were labelled according to the location from which they were obtained and fixed with a 10% formaldehyde solution in separate test tubes. The pathological findings were assessed by an experienced pathologist as benign prostatic hyperplasia, acute or chronic prostatitis, or prostatic intraepithelial neoplasia or carcinoma (Fig. 1d). The grade of the tumour was also evaluated and assigned a standard Gleason score (Table 1).

### Statistical analysis

Student’s *t*-test was used to analyze differences in the quantitative parameters of TICs between benign and malignant lesions (Table 2). Multivariate logistic regression was used to test our first hypothesis: joint analysis of the nodule TICs (e.g. PI in this section) and the normal PZ PI results in better prediction of PCa. We can express the regression model for the probability of malignancy as follows:


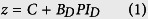



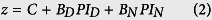



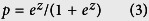


Eqs (1) and (2) are the regression equations calculated for the model using nodule PI only and the nodule and normal PZ PI together. The subscripts *D* and *N* represent the nodule and normal PZ, respectively. *B* and *C* represent the regression coefficient and regression constant, respectively, that correspond to these variables. Subsequently, the *z* values are calculated from Eqs (1) and (2), and the continuous variables are converted from the two categorical dependent variables (benign or malignant) to calculate the probability of malignancy. However, the range of values from positive to negative is large, which makes comparison difficult. Therefore, Eq. (3) represents *Poisson’s* conversion from *z* to the probability of malignancy, *p*, which ranged from 0 to 1. The PI_D_ and PI_N_ values and the significance of these variables in the multivariate logistic regression model are presented in Table 3.

Our second hypothesis was that the improved prediction of PCa results in a significant improvement in diagnostic accuracy in differentiating between benign and malignant nodules. Utilizing the receiver operating characteristic (ROC) curves constructed from malignant probability (*p*) values, we created a standalone PI_D_ regression model based on Eq. (1) and a joint regression model incorporating PI_N_ from Eq. (2) to compare diagnostic accuracy. The differences between areas under the ROC curve were calculated using statistical methods described by DeLong *et al*.^12^ and Hanley *et al*.^13^ Furthermore, a visual assessment of the correlation between nodule PI and normal PZ PI was provided by plotting the benign and malignant nodules with respect to their PI and the corresponding normal PZ PI.

Our third hypothesis was that including normal PZ TICs significantly improves the differentiation of cancer aggressiveness between low-grade and high-grade tumours. Generally, tumours with a Gleason score of 7, 8 or 9 are defined as high-grade tumours, whereas tumours with a Gleason score of 5 or 6 are defined as low-grade tumours^11^,^14^. Using box plot analysis, we established a standalone PI_D_ regression model and incorporated the PI_N_ model into it to compare the probability of malignancy (*p)* and test for significant differences.

All statistical analyses were carried out using SPSS Statistics, version 18 (IBM, Chicago, USA). *P* < 0.05 was considered to indicate statistical significance.

## Results

### Clinical and pathological characteristics

The clinical and pathological characteristics of 132 patients with 134 nodules are listed in Table 1. The results of pathologic examination of the target biopsy samples revealed that there were 54 malignant and 80 benign lesions. Of the 80 benign lesions, 4 nodules were excluded from further analysis as they were identified as chronic prostatitis nodules (n = 4). There was no significant difference between the benign and malignant groups with regard to age, PSA and prostate volume (*p* = 0.475, 0.211 and 0.206, respectively). Among the 54 malignant lesions, four lesions had a Gleason score of 5 (7.4%, 4 of 54); 19 lesions had a Gleason score of 6 (35.2%, 19 of 54); 21 lesions, 7 (38.9%, 21 of 54); seven lesions, 8 (13.0%, 7 of 54); and three lesions, 9 (5.5%, 3 of 54).

### Analysis of the quantitative parameters of TICs

The results of analysis of the quantitative parameters of prostate lesions and normal PZ TICs are shown in Table 2. Total TIC analysis showed that PI (*p* < 0.001), MTT (*p* = 0.011), area under the curve (AUC) (*p* < 0.001) and time from peak to one half (TPH) (*p* = 0.040) were significantly higher in the malignant nodules than in the benign nodules, but AT, wash in slope (WIS) and TTP were not significantly higher (*p* = 0.512, 0.612, and 0.149, respectively; Table 2). Moreover, the TIC parameters of normal PZ tissue were not significantly different between benign and malignant lesions, except for MTT and WIS (*p* = 0.036 and 0.001, respectively; Table 2). Multivariate logistic regression analysis showed that nodule PI (*p* < 0.001), MTT (*p* = 0.014), AUC (*p* < 0.001) and TPH (*p* = 0.041) were significant factors with regard to the prediction of PCa, but AT, WIS and TTP were not significant factors (*p* = 0.509, 0.253 and 0.151, respectively; Table 3).

### The effect of incorporating normal PZ PI, AUC, MTT or TPH in the prediction of PCa

Normal PZ TICs, which reflect the “background” characteristics of prostate tissue, are correlated with the TICs of PZ in prostate nodules. By using logistic regression and Eqs (1) and (2), a regression model using only nodule TIC parameters can be expressed as Eqs (4) and (5), shown below:


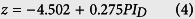



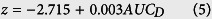


The model incorporating PI_D_ and PI_N_ or AUC_D_ and AUC_N_ can be expressed as Eqs (6) and (7) respectively:


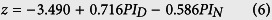



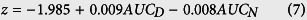


**Figure 2:**
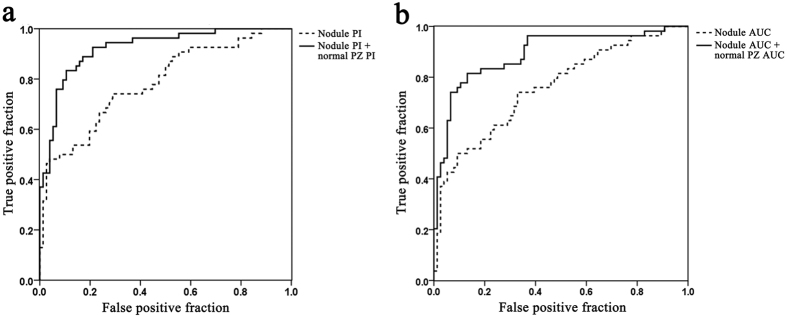
Receiver operating characteristic curve of the regression models based on Eq. (3). The solid line shows the diagnostic accuracy when the model incorporates (**a**) the normal peripheral zone (PZ) peak intensity (PI) in addition to the nodule PIs and (**b**) the normal PZ area under the curve (AUC) in addition to the nodule AUCs. The dotted line shows the diagnostic accuracy of (**a**) nodule PIs only and (**b**) nodule AUCs only.

In both regression models, a dramatic change was introduced by the addition of nodule PI (*p* < 0.001, PI_D_ alone; *p* < 0.001, PI_D_ including PI_N_, Table 3), nodule AUC (*p* < 0.001, AUC_D_ alone; *p* < 0.001, AUC_D_ including AUC_N_, Table 3), normal PZ PI (*p* < 0.001; Table 3) and AUC (*p* < 0.001; Table 3); this significantly improved the prediction of malignancy. Table 4 and Fig. 2a show the ROC curves for the probability value *p* based on the regression models of Eqs (4) and (6): the area under the ROC curve increased by 17.7%, from 0.784 (95% CI, 0.704–0.865) to 0.923 (95% CI, 0.876–0.969) (*p* < 0.001, according to both the DeLong and Hanley methods). Table 4 and Fig. 2b show that the area under the ROC curve based on the regression models of Eqs (5) and (7) increased by 17.5%, from 0.758 (95% CI, 0.673–0.843) to 0.891 (95% CI, 0.832–0.951) (*p* < 0.001, according to both the DeLong and Hanley methods).

However, incorporating the normal MTT or TPH (*p* = 0.285, MTT_N_; *p* = 0.750, TPH_N_; Table 3) failed to improve the prediction accuracy of PCa.

### Diagnostic accuracy of the regression models

Incorporating PI_N_ dramatically improved tumour diagnostic accuracy (*p* < 0.001; PI_D_ only, *p* < 0.001), as did the inclusion of AUC_N_ (*p* < 0.001; AUC_D_ only, *p* < 0.001). When only PI_D_ was incorporated into the regression model, the specificity, sensitivity, positive predictive value (PPV) and negative predictive value (NPV) were 73.7%, 66.7%, 64.3% and 75.7%, respectively; when PI_N_ was also included, these values increased to 90.8% (69 of 76), 79.6% (43 of 54), 86.0% (43 of 50) and 86.3% (69 of 80), respectively. Similarly, when only AUC_D_ was incorporated in the regression model, the specificity, sensitivity, PPV and NPV were 81.6%, 53.7%, 67.4% and 71.3%, respectively; when AUC_N_ was also incorporated, these values increased to 92.1% (70 of 76), 72.2% (39 of 54), 86.7% (39 of 45), and 82.4% (70 of 85), respectively.

### Correlation between nodule TICs and normal PZ TICs

**Figure 3:**
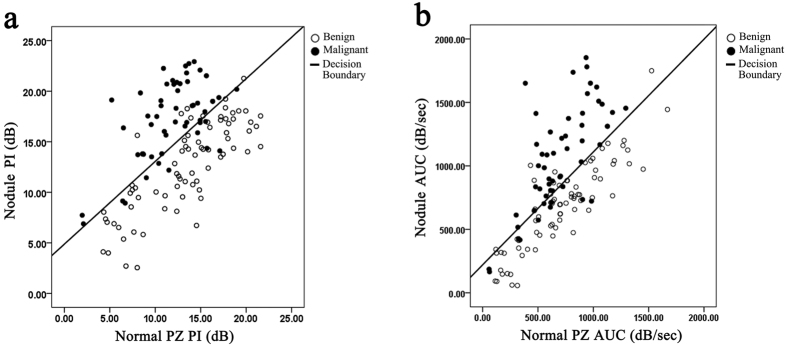
Scatter plots and decision boundary of the (**a**) model incorporating the nodule peak intensity (PI) and the normal peripheral zone (PZ) PI and (**b**) the model incorporating the nodule area under the curve (AUC) and the normal PZ AUC. The line represents the decision boundary; the solid dots, the malignant lesions; and the looped dots, the benign lesions.

In combination with Eq. (3), the regression models established depict the probability of a given nodule being a prostatic malignancy. Based on the models that also incorporate PI_N_ or AUC_N_ (Fig. 3a,b), it seems that a relatively low PI_D_ or AUC_D_ might still be indicative of a highly suspicious tumour if the PI_N_ or AUC_N_ is also low. Thus, the diagnostic accuracy of a threshold based on only the PI_D_ or AUC_D_ value alone may not be very high (decision line in Fig. 3a,b).

### Assessment of cancer aggressiveness based on the regression models

**Figure 4:**
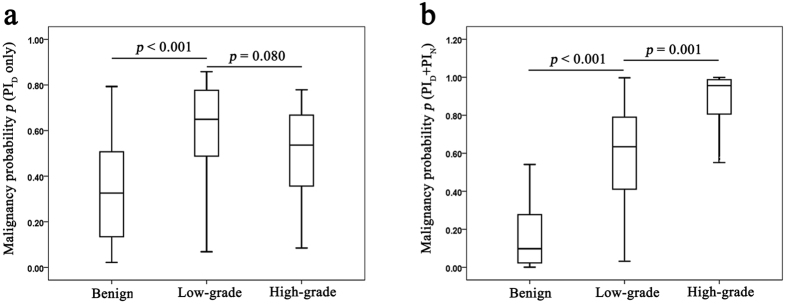
Box plot analysis of the probability of malignancy (*p*) based on (**a**) nodule peak intensity (PI) only and (**b**) normal peripheral zone (PZ) PI in the benign (n = 76), low-grade (n = 23) and high-grade tumour (n = 31) groups.

Based on the probability of malignancy (*p*) of the regression models, it seems that both the PI_D_ only and PI_D_ plus PI_N_ models are significant with regard to differentiating between low-grade tumours and benign lesions (*p* < 0.001 for both, Fig. 4a,b). Moreover, the PI_D_ plus PI_N_ model is significant for differentiating between high-grade tumours and benign lesions (*p* < 0.001, Fig. 4b), but the PI_D_ only model is not (*p* = 0.080, Fig. 4a). Moreover, the inclusion of nodule PI along with normal PZ PI resulted in a significant improvement in the differentiation of high-grade tumours from benign nodules (*p* < 0.001 for both, Fig. 4a,b).

## Discussion

Several studies have reported that CETRUS has high diagnostic sensitivity and specificity for the prediction of PCa^9^,^10^. However, with regard to TICs, the most accurate quantitative parameters differ greatly from each other^9^,^10^, which means that the diagnostic accuracy of the parameters vary. In this study, we found that the perfusion indices PI, MTT, AUC and TPH are significant factors with regard to the prediction of PCa (Tables 2 and 3). However, when the malignancy risk was assessed based on nodule TICs only, there was no evidence to show that any of these factors is significantly better than the others. Moreover, certain other indices that were found to be significant in previous studies on PCa^9^ and liver^15^,^16^ and breast^17^ cancer did not appear significant in our findings, for example, AT (Tables 2 and 3). It is possible that if the inter-patient variation is eliminated or heterogeneity is adjusted for, the diagnostic accuracy of these factors for PCa may improve.

In CETRUS recordings, the entire prostate is represented by groups of data points; moreover, current studies often focus on the PZ hyperperfusion region rather than the whole prostate and do not conduct tissue analysis of other normal tissue^18^,^19^,^20^. Here, we show that incorporating normal tissue TICs could significantly improve the diagnostic accuracy of nodule TICs for PCa detection (Fig. 2a,b). This is consistent with the finding that including background information enhances the discriminative ability of MRI for PCa^11^. Interestingly, the other TIC parameters, such as MTT and TPH, improved the prediction of PCa by themselves, but incorporation of the corresponding normal PZ parameters did not improve PCa prediction (Table 3). This finding indicates that “background” prostate PZ perfusion characteristics affect the “Y-axis” intensity parameters of TICs, such as PI or AUC, but they do not affect the “X-axis” time parameters such as MTT and TPH. Our results also demonstrate that low nodule TIC values are suggestive of a potential malignancy in the presence of low normal PZ TIC values. Thus, it is not feasible to set any standard diagnostic threshold values for nodules or normal parameters (Fig. 3a,b).

The incorporation of PI_D_ plus PI_N_ or AUC_D_ plus AUC_N_ significantly affected the differentiation ability of TICs. However, it not clear whether the TIC values are useful for the precise assessment of cancer aggressiveness, which is commonly estimated by the Gleason score and largely influences PCa management^21^,^22^. Moreover, the contrast-enhanced CETRUS findings for the PCa lesions showed various patterns according to tumour vascularity^23^,^24^ and aggressiveness^25^,^26^; this is indicative of the heterogeneity of this cancer. In our study, incorporating TICs enabled the differentiation of low-grade tumours (n = 23) from high-grade ones (n = 31) (Fig. 4b). This finding indicates that normal PZ TICs play a novel role in the assessment of cancer aggressiveness.

This study had several limitations. Firstly, the use of TICs to assess transition zone tumours has not been investigated. Given that the majority of PCa’s arise in the PZ, this is a major limitation of this study. Secondly, ultrasound-guided prostate biopsy has inevitable false positive and false negative outcomes, which may have affected the final results of our study. Thirdly, the validity of the cancer aggressiveness assessment should be further tested in a large, prospective and multi-cohort study.

In conclusion, our findings demonstrate that incorporating the perfusion characteristics of normal PZ tissue may enable the identification of malignancies based on the quantitative TIC parameters of CETRUS. Thus, this is a novel approach that uses inter-patient differences for PCa prediction and assessment of cancer aggressiveness.

## Additional Information

**How to cite this article**: Huang, H. *et al*. Contrast-enhanced transrectal ultrasound for prediction of prostate cancer aggressiveness: The role of normal peripheral zone time-intensity curves. *Sci. Rep.* **6**, 38643; doi: 10.1038/srep38643 (2016).

**Publisher's note:** Springer Nature remains neutral with regard to jurisdictional claims in published maps and institutional affiliations.

## Figures and Tables

**Table 1:** Clinical and pathologic characteristics of the patients.

Characteristic	Value		
No. of patients	132		
No. of PZ nodules	134		
Clinical characteristics*	Benign lesions** **(n = 80)**	**Malignant lesions (n = 54)**	***p*** **value**
Age (y)	65.9 ± 9.0	67.1 ± 10.8	0.475
PSA (ng/ml)	5.2 ± 3.0	5.9 ± 3.1	0.211
Prostate volume (cm^3^)	50.1 ± 26.5	43.3 ± 13.1	0.206
**Gleason score**	**No. of lesions, n (%)**		
5	4 (7.4)		
6	19 (35.2)		
7	21 (38.9)		
8	7 (13.0)		
9	3 (5.5)		

PZ: peripheral zone, PSA: prostate-specific antigen.

^*^Data are shown as the mean ± standard deviation.

^**^Benign: 76, benign prostatic hyperplasia; 4, chronic prostatitis.

**Table 2:** Quantitative parameters of the nodule and normal peripheral zone time-intensity curves.

Parameters	Nodules (n = 130)			Normal peripheral zone (n = 130)		
	Benign lesions (n = 76)	Malignant lesions (n = 54)	*p* value	Benign lesions (n = 76)	Malignant lesions (n = 54)	*p* value
AT (s)	22.0 ± 6.0	22.7 ± 4.8	0.512	21.7 ± 6.4	21.8 ± 5.7	0.913
PI (dB)	12.6 ± 4.4	17.2 ± 3.9	<0.001	13.4 ± 4.8	11.8 ± 3.6	0.052
MTT (s)	32.3 ± 9.8	37.0 ± 10.4	0.011	30.4 ± 9.5	33.9 ± 9.2	0.036
AUC (dB/s)	685.1 ± 334.1	1055.3 ± 404.4	<0.001	705.5 ± 362.8	699.1 ± 273.3	0.912
TPH (s)	46.5 ± 15.7	52.3 ± 15.8	0.040	44.3 ± 15.0	48.4 ± 14.2	0.119
WIS (dB/s)	1.4 ± 0.6	1.5 ± 0.6	0.612	1.5 ± 0.8	1.1 ± 0.6	0.001
TTP (s)	32.4 ± 8.2	30.5 ± 5.9	0.149	31.7 ± 9.1	33.7 ± 7.3	0.198

AT: arrival time; PI: peak intensity; MTT: mean transit time; AUC: area under the curve; TPH: time from peak to one half; WIS: wash in slope; TTP: time to peak. Values are shown as the mean ± standard deviation.

**Table 3:** Multivariate logistic regression of the quantitative parameters of TICs based on Eqs (1 and 2).

		B_D_		B_N_		C	
Parameters		Value	*p* value	Value	*p* value	Value	*p* value
AT	AT_D_ only	0.021	0.509	—	—	−0.816	0.271
	AT_D_ + AT_N_	0.023	0.508	−0.004	0.903	−0.764	0.369
PI	PI_D_ only	0.275	<0.001	—	—	− 4.502	0.007
	PI_D_ + PI_N_	0.716	<0.001	−0.586	<0.001	− 3.490	0.001
MTT	MTT_D_ only	0.046	0.014	—	—	−1.947	0.004
	MTT_D_ + MTT_N_	0.037	0.076	0.023	0.285	−2.374	0.003
AUC	AUC_D_ only	0.003	<0.001	—	—	−2.715	<0.001
	AUC_D_ + AUC_N_	0.009	<0.001	−0.008	<0.001	−1.985	0.001
TPH	TPH_D_ only	0.024	0.041	—	—	−1.541	0.013
	TPH_D_ + TPH_N_	0.021	0.159	0.005	0.750	−1.629	0.017
WIS	WIS_D_ only	0.156	0.253	—	—	−0.566	0.239
	WIS_D_ + WIS_N_	0.479	0.163	−1.043	0.001	0.295	0.595
TTP	TTP_D_ only	−0.036	0.151	—	—	0.802	0.323
	TTP_D_ + TTP_N_	−0.056	0.050	0.045	0.061	−0.061	0.948

AT: arrival time; PI: peak intensity; MTT: mean transit time; AUC: area under the curve; TPH: time from peak to one half; WIS: wash in slope; TTP: time to peak.

D = nodule peripheral zone tissue, N = normal peripheral zone tissue, B = regression coefficient, C = regression constant.

**Table 4:** Diagnostic performance of the nodule parameters only and both the nodule parameters and normal peripheral zone PI or AUC.

Parameters		Sensitivity (%)	Specificity (%)	PPV (%)	NPV (%)	AUC (%)
PI	PI_D_ only	66.7 (36/54)	73.7 (56/76)	64.3 (36/56)	75.7 (56/74)	78.4 (70.4–86.5)
	PI_D_ + PI_N_	79.6 (43/54)	90.8 (69/76)	86.0 (43/50)	86.3 (69/80)	92.3 (87.6–96.9)
AUC	AUC_D_ only	53.7 (29/54)	81.6 (62/76)	67.4 (29/43)	71.3 (62/87)	75.8 (67.3–84.3)
	AUC_D_ + AUC_N_	72.2 (39/54)	92.1 (70/76)	86.7 (39/45)	82.4 (70/85)	89.1 (83.2–95.1)

PI: peak intensity, AUC: area under the curve, PPV: positive predictive value, NPV: negative predictive value. D = nodule peripheral zone tissue, N = normal peripheral zone tissue.

The AUC values are followed by the 95% CIs in parentheses.
